# A spatial-epidemiological dataset of subjects infected by SARS-CoV-2 during the first wave of the pandemic in Mashhad, second-most populous city in Iran

**DOI:** 10.1186/s13104-021-05710-9

**Published:** 2021-07-27

**Authors:** Shahab MohammadEbrahimi, Alireza Mohammadi, Robert Bergquist, Mahsan Akbarian, Mahnaz Arian, Elahe Pishgar, Behzad Kiani

**Affiliations:** 1 Department of Medical Informatics, School of Medicine, Mashhad University of Medical Sciences, Mashhad, Iran; 2 Student Research Committee, Mashhad University of Medical Sciences, Mashhad, Iran; 3 Department of Geography and Urban Planning, Faculty of Social Sciences, University of Mohaghegh Ardabili, Ardabil, Iran; 4 Ingerod, Brastad, Sweden (formerly with the UNICEF/UNDP/World Bank/WHO Special Program for Research and Training in Tropical Diseases, World Health Organization), Geneva, Switzerland; 5 Department of Physiology, School of Medicine, Mashhad University of Medical Sciences, Mashhad, Iran; 6 Department of Infectious Diseases, School of Medicine, Mashhad University of Medical Sciences, Mashhad, Iran; 7 Department of Human Geography, Faculty of Earth Science, Shahid Beheshti University, Tehran, Iran

**Keywords:** COVID-19, SARS-CoV-2, Spatial epidemiology, Geographical information systems, Iran

## Abstract

**Objective:**

In March 2020, Iran tackled the first national wave of COVID-19 that was particularly felt in Mashhad, Iran’s second-most populous city. Accordingly, we performed a spatio-temporal study in this city to investigate the epidemiological aspects of the disease in an urban area and now wish to release a comprehensive dataset resulting from this study.

**Data description:**

These data include two data files and a help file. Data file 1: “COVID-19_Patients_Data” contains the patient sex and age + time from symptoms onset to hospital admission; hospitalization time; co-morbidities; manifest symptoms; exposure up to 14 days before admission; disease severity; diagnosis (with or without RT-PCR assay); and outcome (recovery vs. death). The data covers 4000 COVID-19 patients diagnosed between 14 Feb 2020 and 11 May 2020 in Khorasan-Razavi Province. Data file 2: “COVID-19_Spatiotemporal_Data” is a digital map of census tract divisions of Mashhad, the capital of the province, and their population by gender along with the number of COVID-19 cases and deaths including the calculated rates per 100,000 persons. This dataset can be a valuable resource for epidemiologists and health policymakers to identify potential risk factors, control and prevent pandemics, and optimally allocate health resources.

## Objective

A novel respiratory infection named coronavirus disease-2019 (COVID-19) originated in November 2019 and produced a major outbreak globally [1]. The pathogen was diagnosed as one of the coronavirus family and is currently known as the Severe Acute Respiratory Syndrome Coronavirus-2 (SARS-CoV-2) [2]. Due to the highly contagious nature of this virus, it spread in a short time to almost all countries and eventually being declared a pandemic by the World Health Organization (WHO) on 11 March 2020 [3, 4]. As of 19 July 2021, more than 191 million confirmed cases and approximately 2.15% mortality were identified worldwide. Iran's share at that time was close to 3,523,000 cases with more than 87,000 deaths [5]. Analysing and interpreting the spatio-temporal transmission patterns of the virus are indispensable in order to generate the best-tailored strategies [6]. The strength of Geographic Information Systems (GIS) applications lies in their capability of mapping geographical disease distributions thereby visualizing trends of their spread, which can be utilized for modelling spatial aspects of disease occurrence in relation to the ambient environments [7–9].

The spatial patterns of COVID-19 incidence were investigated by census level with addressing the epidemiological features, during the first wave of the pandemic in Mashhad, Iran [10]. In that study, hotspots and high/low-risk areas were detected by using the Getis-Ord Gi* and Local Moran’s *I* statistic [11, 12]. A univariate regression model was developed to quantify the association of COVID-19 mortality with common risk factors [13] including age [14, 15], sex [16, 17], co-morbidities [18, 19], hospitalization length [20, 21] and transfer to an Intensive Care Unit (ICU) [16, 20]. Here, a comprehensive spatial-epidemiological dataset linked to other urban data at the census level is offered for further investigation to identify transmission trends and clustering patterns of the COVID-19 incidence in the densely populated city.

## Data description

In the current study, the COVID-19 data were collected in the referral hospitals and health-care centres under the supervision of Mashhad University of Medical Sciences (MUMS). These data were related to all people infected by SARS-CoV-2 in Khorasan-Razavi Province (KRP) and they covered three months with the start coinciding with the beginning of the COVID-19 outbreak in KRP (i.e. from 14 Feb to 11 May 2020). The data included 4000 people referred to the health-care centres and hospitals due to COVID-19 infection with cases either confirmed clinically (n = 2675) or by laboratory tests (n = 1325) using the Reverse Transcription Polymerase Chain Reaction (RT-PCR) assay. Demographic data of all neighbourhoods came from census blocks statistics of 2018–2019 [22].

Addresses of patients in the city of Mashhad with confirmed COVID-19 by RT-PCR test (n = 727) were geocoded manually using the Google MyMaps software (http://www.google.com/mymaps). Mashhad is the capital city of KRP and is the second-most populous city in Iran which has 1,301 census tracts. Five age groups, including 0–14, 15–24, 25–44, 44–64, and > 65 years old, were used to calculate the age- and sex-adjusted incidence and death rates of COVID-19 in each census tract. In order to avoid the identity of participating COVID-19 cases, the point-density data, expressing patients' physical addresses, were aggregated into each census tract and stratified into ten-day intervals during the study period.

Table 1 shows details of the two data files, a help file and access links. Data file 1 covers the demographic and clinical information of 4000 COVID-19 cases in Excel file format (*.xlsx). Each data row includes patient sex and age + time from symptoms onset to hospital admission; hospitalization time; co-morbidities; manifest symptoms; exposure up to 14 days before admission; disease severity; diagnosis (with or without RT-PCR assay); and outcome (recovery vs. death). Data file 2 covers the spatio-temporal data of those infected by SARS-CoV-2 including polygon shape-files (*.shp) representing the location of all COVID-19 cases aggregated at the census tract level. This data file covers the following information: an identification code of each census; the total population as well as the population by gender in each census; the number of cases and deaths by COVID-19 along with the calculated rates per 100,000 persons separately for each census; and the number of cases and deaths due to COVID-19 based on ten-day intervals. File 3 is a help file for both data files which represents the name and description of each field. Since Mashhad is located at 36° N, 59° E, the projection system of WGS_1984_UTM_Zone_40N was used as the Projected Coordinate System (PCS) for all GIS layers. Due to the need to find better preventive measures and improve hospital care in response to the irreversible psychological and physical effects of COVID-19 [23–25], the data in the current study can be used as a basis for spatial modelling of the disease providing reliable knowledge to other researchers in various fields such as health geography, urban policymaking and healthcare research.

**Table 1:** Overview of data sets

Label	Name of data file	File types (Extensions)	Data repository and identifier (DOI or accession number)
Data file 1	COVID-19_Patients_Data	Excel file (*.xlsx)	*Harvard Dataverse* https://doi.org/10.7910/DVN/SSJ7WT [26]
Data file 2	COVID-19_Spatiotemporal_Data	Shape file (*.shp)	*Harvard Dataverse* https://doi.org/10.7910/DVN/SSJ7WT [26]
Data file 3	Help file	Excel file (*.xlsx)	*Harvard Dataverse* https://doi.org/10.7910/DVN/SSJ7WT [26]

## Limitations

Only 33% of all included cases were confirmed by RT-PCR testing, the rest of the cases were clinically approved. Separate data about hypertension and recovered cases with remaining disease complications (long COVID) were not collected. Due to the short period covered (3 months), the capabilities of spatial analysis cannot tell us more now. For the future, it is suggested to study the spatial and temporal dynamics of the disease over a longer period in order to provide more operational solutions.

## Data Availability

The data described in this Data note can be freely and openly accessed on the Harvard Dataverse under (https://doi.org/10.7910/DVN/SSJ7WT) [26]. Please see Table 1 and the reference list for details and links to the data.

## Abbreviations

COVID-19: Corona Virus Disease-2019
SARS-CoV-2: Severe Acute Respiratory Syndrome Coronavirus-2
GIS: Geographic Information System
KDE: Kernel Density Estimation
LOS: Length of Stay
ICU: Intensive Care Unit
MUMS: Mashhad University of Medical Sciences
KRP: Khorasan-Razavi Province
RT-PCR: Reverse Transcription Polymerase Chain Reaction
KML: Keyhole Markup Language
PCS: Projected Coordinate System
